# Dysregulation of choline metabolism and therapeutic potential of citicoline in Huntington's disease

**DOI:** 10.1111/acel.14302

**Published:** 2024-08-14

**Authors:** Kuo‐Hsuan Chang, Mei‐Ling Cheng, Hsiang‐Yu Tang, Chung‐Yin Lin, Chiung‐Mei Chen

**Affiliations:** ^1^ Department of Neurology, Chang Gung Memorial Hospital‐Linkou Medical Center Chang Gung University School of Medicine Taoyuan Taiwan; ^2^ Department of Biomedical Sciences Chang Gung University Taoyuan Taiwan; ^3^ Metabolomics Core Laboratory, Healthy Aging Research Center Chang Gung University Taoyuan Taiwan; ^4^ Clinical Metabolomics Core Core Laboratory Chang Gung Memorial Hospital at Linkou Taoyuan Taiwan; ^5^ Institute for Radiological Research Chang Gung University Taoyuan Taiwan

**Keywords:** choline, citicoline, glycerophosphocholine phosphodiesterase 1, Huntington's disease

## Abstract

Huntington's disease (HD) is associated with dysregulated choline metabolism, but the underlying mechanisms remain unclear. This study investigated the expression of key enzymes in this pathway in R6/2 HD mice and human HD postmortem brain tissues. We further explored the therapeutic potential of modulating choline metabolism for HD. Both R6/2 mice and HD patients exhibited reduced expression of glycerophosphocholine phosphodiesterase 1 (GPCPD1), a key enzyme in choline metabolism, in the striatum and cortex. The striatum of R6/2 mice also showed decreased choline and phosphorylcholine, and increased glycerophosphocholine, suggesting disruption in choline metabolism due to GPCPD1 deficiency. Treatment with citicoline significantly improved motor performance, upregulated anti‐apoptotic Bcl2 expression, and reduced oxidative stress marker malondialdehyde in both brain regions. Metabolomic analysis revealed partial restoration of disrupted metabolic patterns in the striatum and cortex following citicoline treatment. These findings strongly suggest the role of GPCPD1 deficiency in choline metabolism dysregulation in HD. The therapeutic potential of citicoline in R6/2 mice highlights the choline metabolic pathway as a promising target for future HD therapies.

## Graphical abstract

Mutant huntingtin inhibits expression of glycerophosphocholine phosphodiesterase 1 (GPCPD1) in the striatum and cortex of both R6/2 mice and HD patients. It also decreases the choline kinase alpha (CHKA) expression in the striatum of R6/2 mice. The GPCPD1 deficiency leads to reduced choline and phosphocholine, and increased glycerophosphocholine in the striatum of R6/2 mice, suggesting disruption in choline metabolism. The reduced choline, phosphocholine, and CDP‐choline (citicoline) cause increased oxidative stress (malondialdehyde) and apoptosis (Bcl2), disturbed metabolism, and impaired motor performance, all of which can be rescued by citicoline treatment in R6/2 mice.

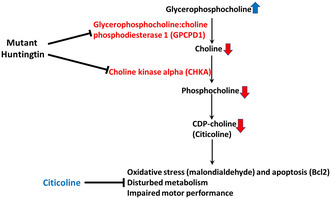

**Abbreviations**
ACTB: beta‐actin
Actb: beta‐actin
BCL2: B cell lymphoma 2
BSA: bovine serum albumin
CCT: CTP: phosphocholine cytidyltransferase
CHKA: choline kinase alpha
CHKB: choline kinase beta
CHPT1: CDP‐choline:1,2‐diacylglycerolphosphocholine transferase 1
CSF: cerebrospinal fluid
CT: cycle threshold
GPCPD1: glycerophosphocholine phosphodiesterase 1
HD: Huntington's disease
HTT: huntingtin
MDA: malondialdehyde
MRS: magnetic resonance spectroscopy
NC: normal control
NGS: normal goat serum
NRF2: nuclear factor erythroid 2‐related factor 2
PB: phosphate buffer
PBS: phosphate‐buffered saline
PINK1: phosphatase and tensin homolog‐induced kinase 1
PLA2: phospholipase A2
PLD1: phospholipase D1
PolyQ: polyglutamine
PPAP2A: phosphatidic acid phosphatase 2a
preHD: presymptomatic Huntington's disease
PRKN: parkin RBR E3 ubiquitin protein ligase
qRT‐PCR: quantitative real‐time polymerase chain reaction
SIRT1: sirtuin 1
UPLC‐MS/MS: Ultra‐performance liquid chromatography–mass spectrometry/mass spectrometry
UPLC‐Q‐TOF‐MS: ultra‐performance liquid chromatography‐quadrupole/time‐of‐flight mass spectrometry
WT: wide‐type

## 1 INTRODUCTION

Huntington's disease (HD) is a rare neurodegenerative disease characterized by cognitive decline, choreiform movements, and various psychiatric manifestations (Medina et al., 2022). It is caused by the expansion of CAG trinucleotide repeats, which encode a polyglutamine (polyQ) tract in exon 1 of the huntingtin (*HTT*) gene (MacDonald et al., 1993). PolyQ expansion leads to structural alterations in the huntingtin protein, resulting in the formation of aggregates within the nucleus and cytoplasm (Tabrizi et al., 2020). These aggregates disrupt cellular metabolism and mitochondrial function, which significantly contributes to the development of this disease (Tabrizi et al., 2020). Evidence from HD patients and transgenic mouse models suggests that metabolic disturbances occur early in the disease process (Andrews & Brooks, 1998; Cheng et al., 2016; Lowe et al., 2022; Mangiarini et al., 1996; Saft et al., 2005; Young, 2003). HD patients and presymptomatic HD (preHD) carriers exhibit weight loss despite consistent food intake (Mangiarini et al., 1996; Young, 2003). Positron emission tomography and magnetic resonance spectroscopy have revealed decreased metabolic rates in distinct brain regions, including the caudate, putamen, frontal, and parietal cortex, in both HD patients and preHD carriers (Andrews & Brooks, 1998). Disturbances in energy metabolism have also been detected in the skeletal muscles of both HD patients and preHD carriers (Saft et al., 2005). This accumulating evidence strongly supports the pivotal role of dysregulated metabolic processes in the pathogenesis of HD.

Choline is a precursor of important cell components and signaling molecules, including phosphatidylcholine, lysophosphatidylcholine, choline plasmalogen, and sphingomyelin, all of which play essential roles in cell membrane formation and cellular function (Zeisel et al., 1991). Studies have shown that HD patients and transgenic mouse models exhibit disruptions in choline metabolism (Carroll et al., 2015; Cheng et al., 2016; Gomez‐Anson et al., 2007; Manyam et al., 1990; McGarry et al., 2020; Padowski et al., 2014; Tsang et al., 2009; van den Bogaard et al., 2014). Phosphatidylcholine and sphingomyelin accumulate in the striatum of *Hdh* ^*Q111*^ knockin mice (Carroll et al., 2015), while reduced phosphatidylcholine levels have been observed in the frontal cortex of the 3‐nitropropionic acid‐induced mouse model (Tsang et al., 2009). A significantly lower abundance of phosphatidylcholine was found in postmortem cortex and putamen of HD patients (Phillips et al., 2022). HD patients exhibit decreased choline levels in the cerebrospinal fluid (CSF) (Manyam et al., 1990), and preHD carriers exhibit lower choline levels in the frontal cortex, as determined by proton magnetic resonance spectroscopy (MRS) (Gomez‐Anson et al., 2007). Another MRS study revealed a notable decrease in choline levels in the putamen of HD patients over the course of 2 years (van den Bogaard et al., 2014). The choline levels in the caudate are correlated with volume of the caudate in HD patients (Padowski et al., 2014). Additionally, we have shown reduced plasma phosphatidylcholine levels in HD patients (Cheng et al., 2016). While our findings suggest altered choline metabolism in both brain and peripheral blood of HD patients, the regulatory mechanisms behind these changes and the potential therapeutic efficacy of choline supplementation remain unclear.

Given the involvement of phosphatidylcholines and choline in HD pathogenesis, we investigated the mechanism underlying impaired choline metabolism in the brains of R6/2 HD mice and human HD patients. We also explored the neuroprotective potential of citicoline, a compound that can be converted into phosphatidylcholine, in R6/2 mice. Our results advance our understanding of HD pathogenesis and pave the way for effective therapies by targeting choline metabolism.

## 2 RESULTS

### 2.1 Enzyme expression in the choline metabolism pathway in HD mouse brains

**FIGURE 1:**
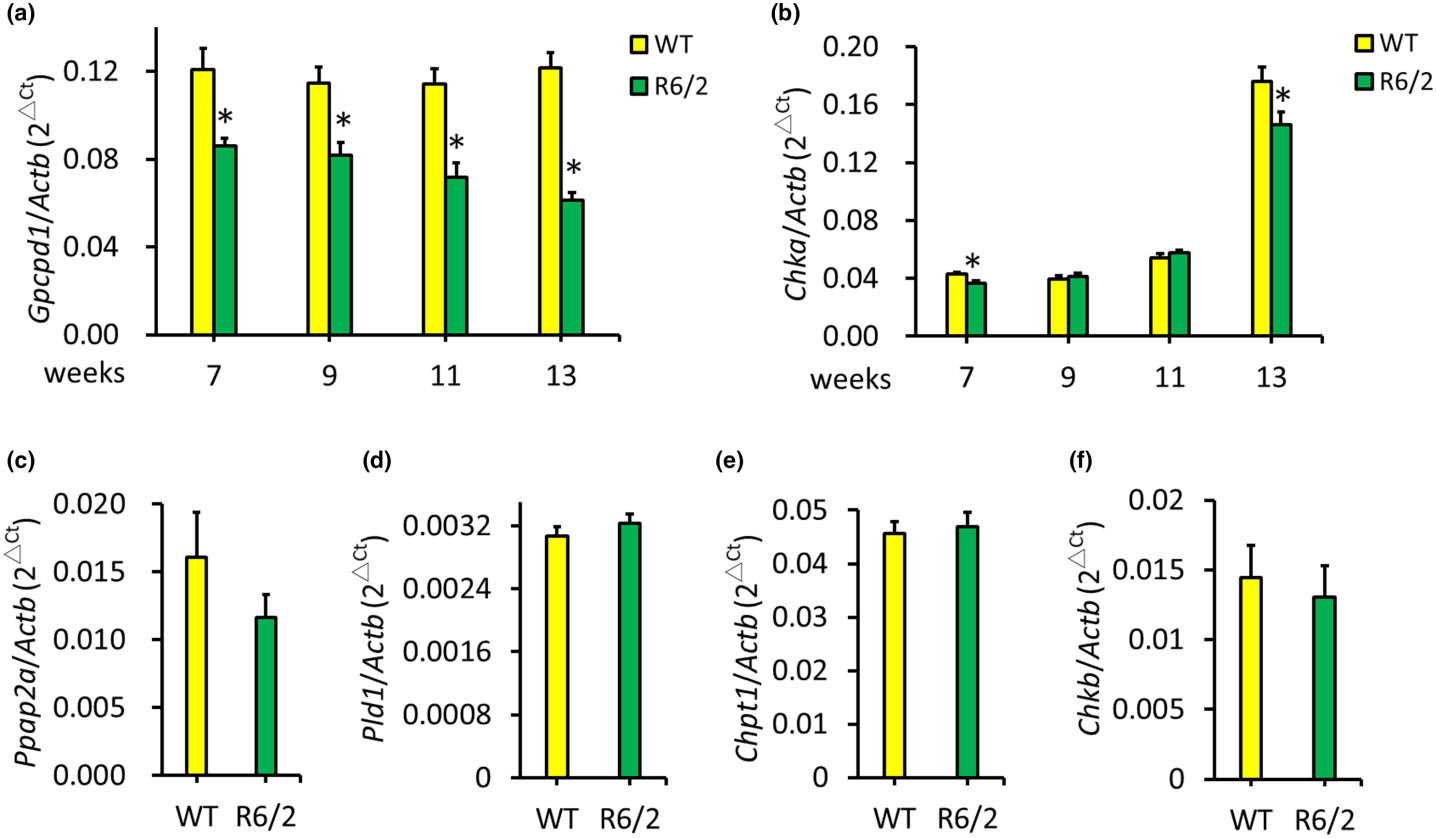
mRNA expression levels of enzymes involved in choline metabolism in the striatum of R6/2 HD mice. Gpcpd1 (a) and Chka (b) mRNA levels in the striatum of R6/2 mice (*n* = 14–20) compared with those in wild‐type (WT) littermates (*n* = 14–20) at 7–13 weeks of age. *Ppap2a* (c), *Pld1* (d), *Chpt1* (e), and *Chkb* (f) mRNA expression levels in the striatum of R6/2 mice (*n* = 20) compared with those in the striatum of WT littermates (*n* = 20) at 13 weeks of age. The expression levels were normalized to those of β‐Actin (Actb). *p < 0.05, R/6 versus WT.

**FIGURE 2:**
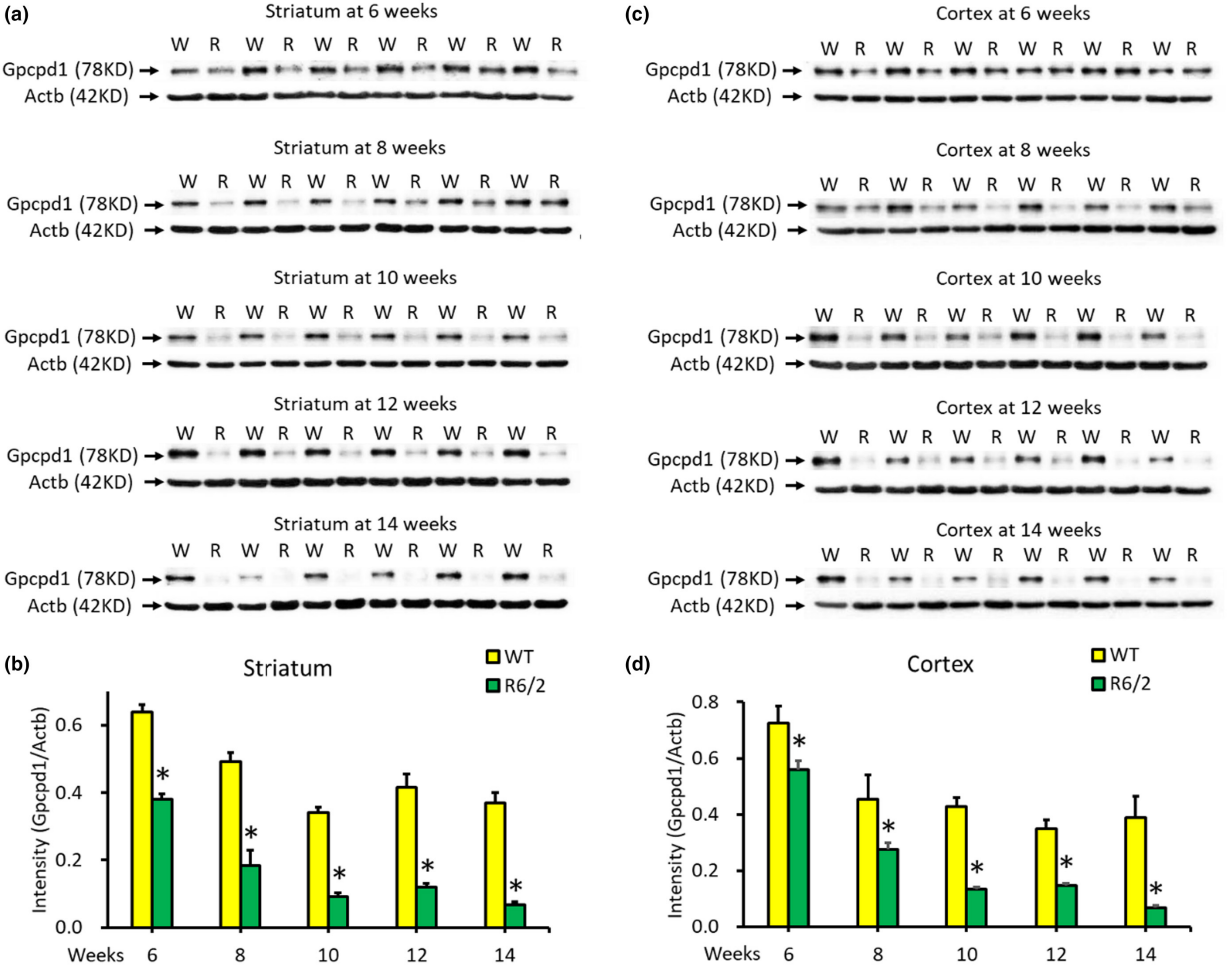
Gpcpd1 Protein levels in the striatum and cortex of R6/2 HD mice at different ages (6–14 weeks). Western blot analysis of Gpcpd1 protein expression in the striatum (a, b) and cortex (c, d) of R6/2 mice (*n* = 6) compared with that in wild‐type (WT) littermates (*n* = 6) at 6–14 weeks of age. W indicates wild‐type littermates, and R indicates R6/2 mice. The expression levels were normalized to that of β‐Actin (Actb). *p < 0.05, R6/2 versus WT.

Given that altered levels of choline metabolism metabolites have been demonstrated in the plasma of HD (Cheng et al., 2016), we examined the gene expression of enzymes involved in the choline metabolism pathway (Figure 7) including *Gpcpd1*, *Chka*, *Chkb*, *Ppap2a*, *Pld1*, and *Chpt1*, in the brains of R6/2 transgenic HD mice. We found a significant reduction in the expression level of *Gpcpd1* in the striatum from 7 to 13 weeks of age compared to that in the striata of WT littermates (Figure 1a). The expression of *Chka* was downregulated in the striatum of R6/2 mice at 7 and 13 weeks of age, but no significant differences were observed between R6/2 mice and WT littermates at 9 and 11 weeks of age (Figure 1b). The expression levels of the remaining genes in this pathway, *Ppap2a*, *Pld1*, *Chpt1*, and *Chkb*, were unchanged in the R6/2 mice compared to those in the WT mice at 13 weeks of age (Figure 1c–f). Subsequently, we examined the protein levels of Gpcpd1 in the striatum and cortex of R6/2 mice. The results showed significantly lower Gpcpd1 protein levels in both the striatum and cortex of R6/2 mice compared with their WT littermates from 6 to 14 weeks of age (Figure 2a–d). These findings align with previous observations of altered metabolites in the choline metabolism pathway in HD patients (Cheng et al., 2016).

### 2.2 Metabolite analysis of the choline metabolism pathway in HD mouse brains

**FIGURE 3:**
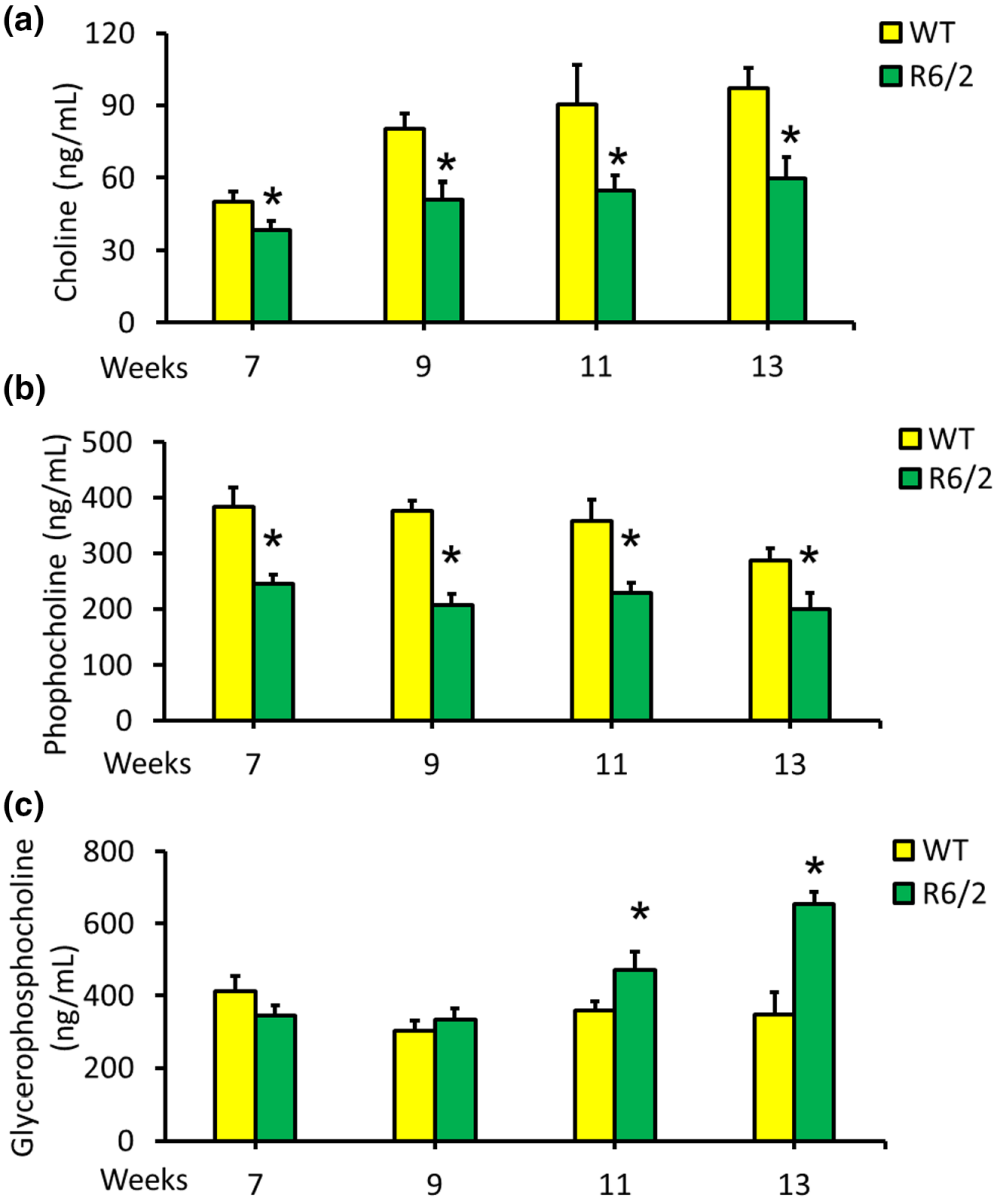
Changes in the choline metabolism of R6/2 transgenic mice at different ages (7–13 weeks). The levels of choline (a), phosphocholine (b), and glycerophosphocholine (c) in the striatum of R6/2 mice (*n* = 20) compared with those in wild‐type (WT) littermates (*n* = 20) at 7–13 weeks of age. *p < 0.05, R6/2 versus WT.

Gpcpd1 catalyzes the conversion of glycerophosphocholine to choline and phosphocholine. Given the reduced levels of Gpcpd1 in the brains of R6/2 mice, we investigated the levels of its downstream metabolites, choline and phosphocholine, as well as the upstream metabolite glycerophosphocholine (Corda et al., 2014), in the striatum of R6/2 mice. We found significant decreases in choline and phosphocholine levels in the striatum of R6/2 mice compared to those in the striatum of WT mice from 7 to 13 weeks of age (Figure 3a,b). However, compared with those in littermates, glycerophosphocholine levels in the striatum of R6/2 mice were significantly greater at 11 to 13 weeks of age (Figure 3c). These findings suggest that the reduced enzymatic activity of GPCPD1 in glycerophosphocholine metabolism leads to decreased choline and phosphorylcholine levels in the striatum of R6/2 mice.

### 2.3 Reduced expression of GPCPD1 in HD postmortem brains

**FIGURE 4:**
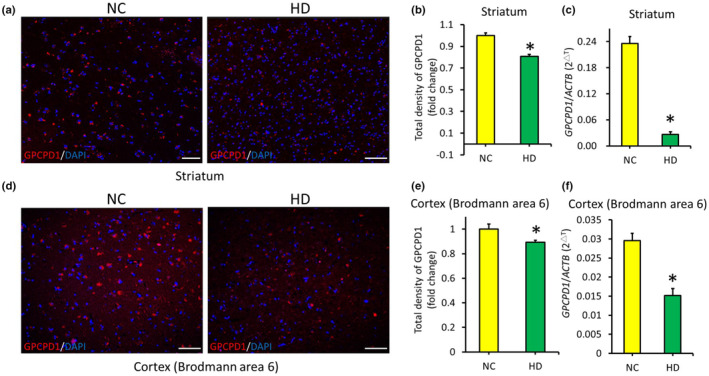
GPCPD1 expression in postmortem brain tissues from Huntington's disease patients. Immunohistochemical analysis of GPCPD1 in the striatum (a, b) and cortex (d, e) of patients with Huntington’ s disease (HD, *n* = 10) compared with that in normal controls (NCs, *n* = 7). All the data were normalized to the intensity of the DAPI‐stained cells and the NC group. Scale bar: 150 μm. GPCPD1 mRNA levels in the striatum (c) and cortex (f) of HD patients (*n* = 10) compared with those in the NC group (*n* = 1, triplicate). *p < 0.05, HD versus NC.

We obtained brain tissue blocks and section slides from the striatum and cortex (Brodmann area 6, BA6) of 10 HD patients and 7 control subjects. Immunohistochemistry revealed lower GPCPD1 protein expression in both the striatum (Figure 4a,b) and cortex (Figure 4d,e) of HD patients than in those of controls. Consistent with these findings, qRT–PCR demonstrated that *GPCPD1* was downregulated in both the striatum (Figure 4c) and cortex (Figure 4f) of HD patients. These results confirmed that GPCPD1 was downregulated in the brains of individuals with HD.

### 2.4 The effects of drugs targeting the choline metabolism pathway in HD mice

**FIGURE 5:**
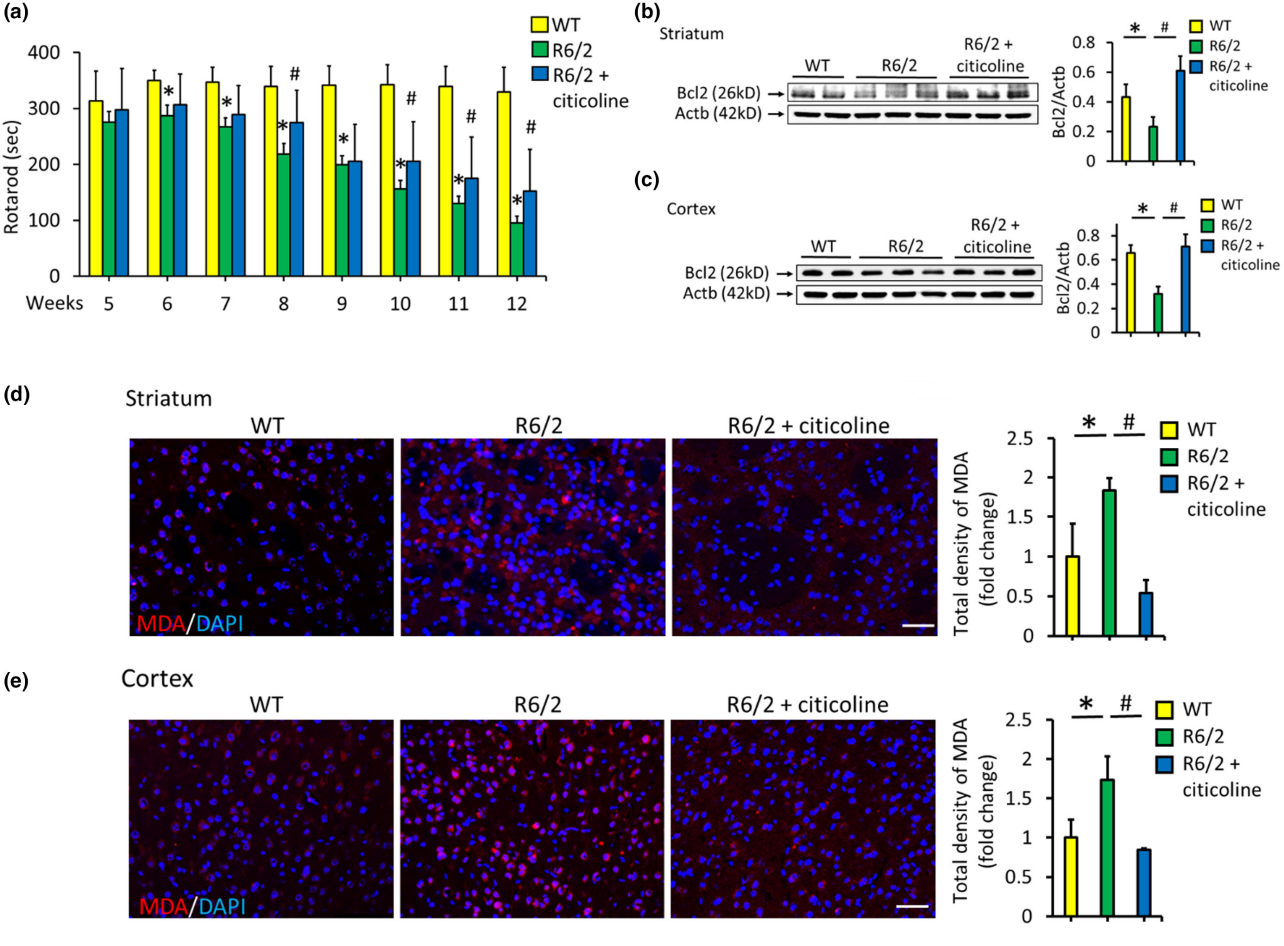
Citicoline improved motor performance and reduced oxidative stress in R6/2 Huntington's disease mice. (a) Rotarod test of R6/2 mice treated with (*n* = 20) or without (*n* = 20) citicoline from 5 to 12 weeks of age compared with wild‐type (WT) littermates. Representative western blot showing Bcl2 protein expression in the striatum (b) and cortex (c) of 12‐week‐old R6/2 mice treated with (*n* = 5) or without citicoline (*n* = 5) compared to that in WT littermates. Representative immunohistochemical images of malondialdehyde (MDA) in the striatum (d) and cortex (e) of 12‐week‐old R6/2 mice treated with (*n* = 4) or without citicoline (*n* = 4) compared to those of WT littermates. All the data were normalized to the intensity of DAPI staining and the intensity of the WT. Scale bar: 150 μm. *p < 0.05, R6/2 versus WT. #*p* < 0.05, R6/2 + citicoline vs R6/2.

**FIGURE 6:**
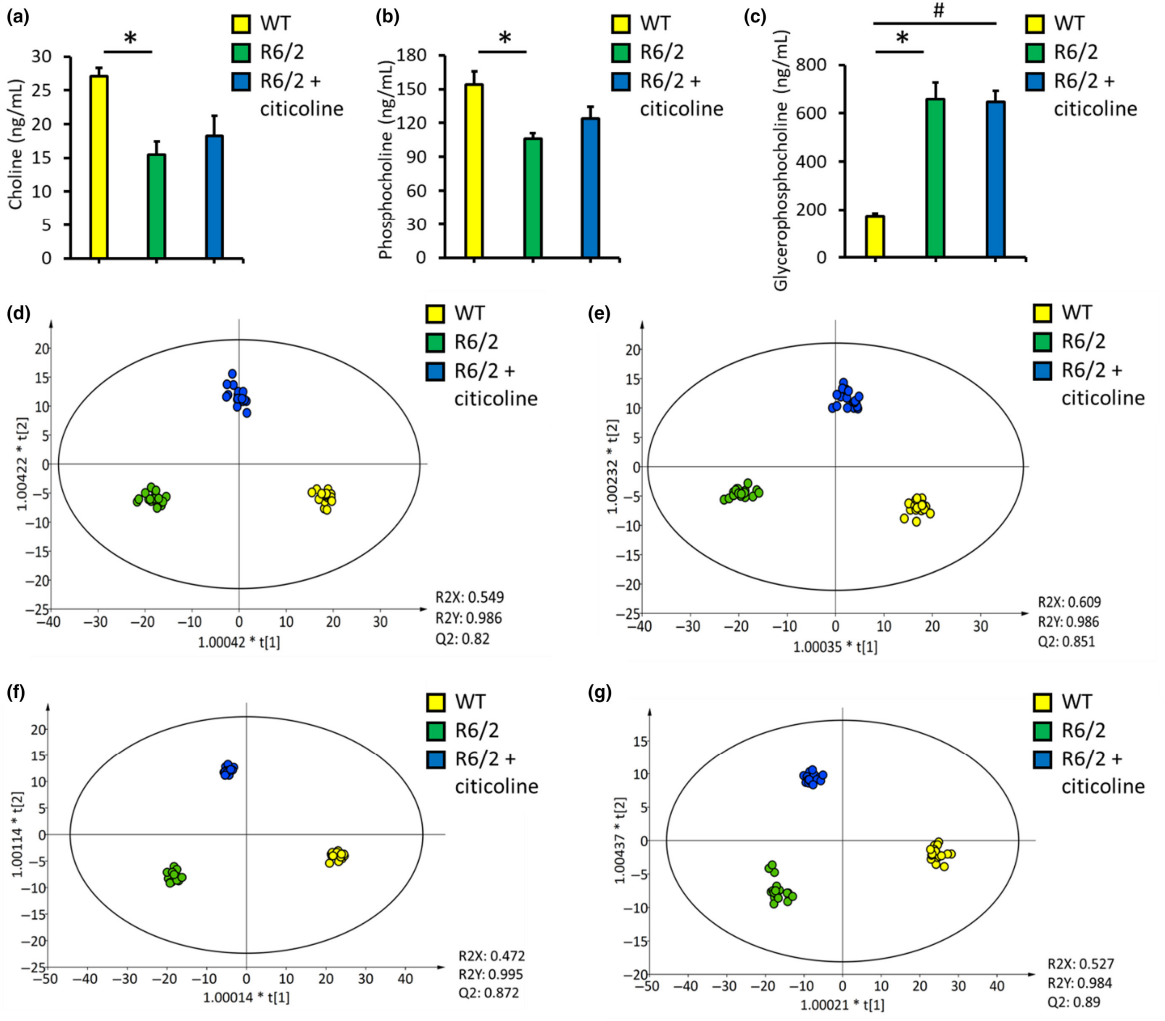
Metabolic changes in the brains of R6/2 transgenic mice following citicoline treatment. Choline (a), phosphocholine (b), and glycerophosphocholine (c) levels in the striatum of 12‐week‐old R6/2 mice treated with (*n* = 6) or without citicoline (*n* = 6) compared with wild‐type (WT) littermates. Orthogonal partial least squares discriminant analysis (OPLS‐DA) of lipidomics in positive (d) and negative (e) ion modes of the striatum of 12‐week‐old R6/2 mice treated with (*n* = 6) or without citicoline (*n* = 6), compared with those from WT littermates. OPLS‐DA of lipidomics in the positive (f) and negative (g) ion modes of the cortex of 12‐week‐old R6/2 mice treated with (*n* = 6) or without citicoline (*n* = 6), compared with those from WT littermates. *p < 0.05, R6/2 versus WT. #*p* < 0.05, R6/2 + citicoline versus WT.

The reduced levels of choline and phosphorylcholine suggest the potential of choline administration as a treatment for HD. Citicoline, also referred to as cytidine diphosphocholine, is rapidly absorbed and metabolized into choline and cytidine (Lopez et al., 1987), making it a potential source of choline. Citicoline has shown lower toxicity than choline (Synoradzki & Grieb, 2019) and is capable of ameliorating metabolic deficits in rats with brain ischemia (Hurtado et al., 2005). Therefore, we examined the potential therapeutic effect of citicoline in R6/2 mice. Consistent with previous reports (K. H. Chang et al., 2015), R6/2 mice showed a decrease in body weight between 11 and 12 weeks. Their blood sugar levels were elevated between 9 and 12 weeks (Figure S1), suggesting a metabolic disturbance in HD pathogenesis (Montojo et al., 2017). Citicoline administration did not influence either body weight or blood sugar in R6/2 mice. Compared with WT mice, R6/2 mice displayed a progressive decline in rotarod performance from 6 to 12 weeks of age (Figure 5a). Citicoline administration significantly improved rotarod performance at 8, 10, 11, and 12 weeks of age. The expression of the negative regulator of apoptosis Bcl2 was significantly downregulated in both the striatum and cortex of R6/2 mice at 12 weeks of age (Figure 5b,c), whereas citicoline treatment upregulated this expression in both the striatum and cortex (Figure 5b,c). Malondialdehyde (MDA), a marker of lipid peroxidation that triggers cell death (Del Rio et al., 2005), was significantly elevated in both the striatum and cortex of R6/2 mice compared to WT mice at 12 weeks of age (Figure 5d,e). Citicoline treatment significantly reduced MDA levels in both brain regions. However, the striatal levels of choline, phosphorylcholine, and glycerophosphocholine remained unchanged after citicoline treatment (Figure 6a–c). Metabolomic profiling revealed differences in positive and negative ionization in the striatum between R6/2 and WT mice, with citicoline partially restoring the metabolic pattern in R6/2 mice toward that of WT mice (Figure 6d,e). Similarly, the metabolic profile in the cortex of R6/2 mice was markedly distinct from that in the cortex of WT mice (Figure 6f,g), and citicoline treatment partially shifted the metabolic pattern closer to that of WT mice. These results suggest the potential therapeutic benefit of citicoline in HD, possibly through the normalization of metabolic patterns.

## 3 DISCUSSION

**FIGURE 7:**
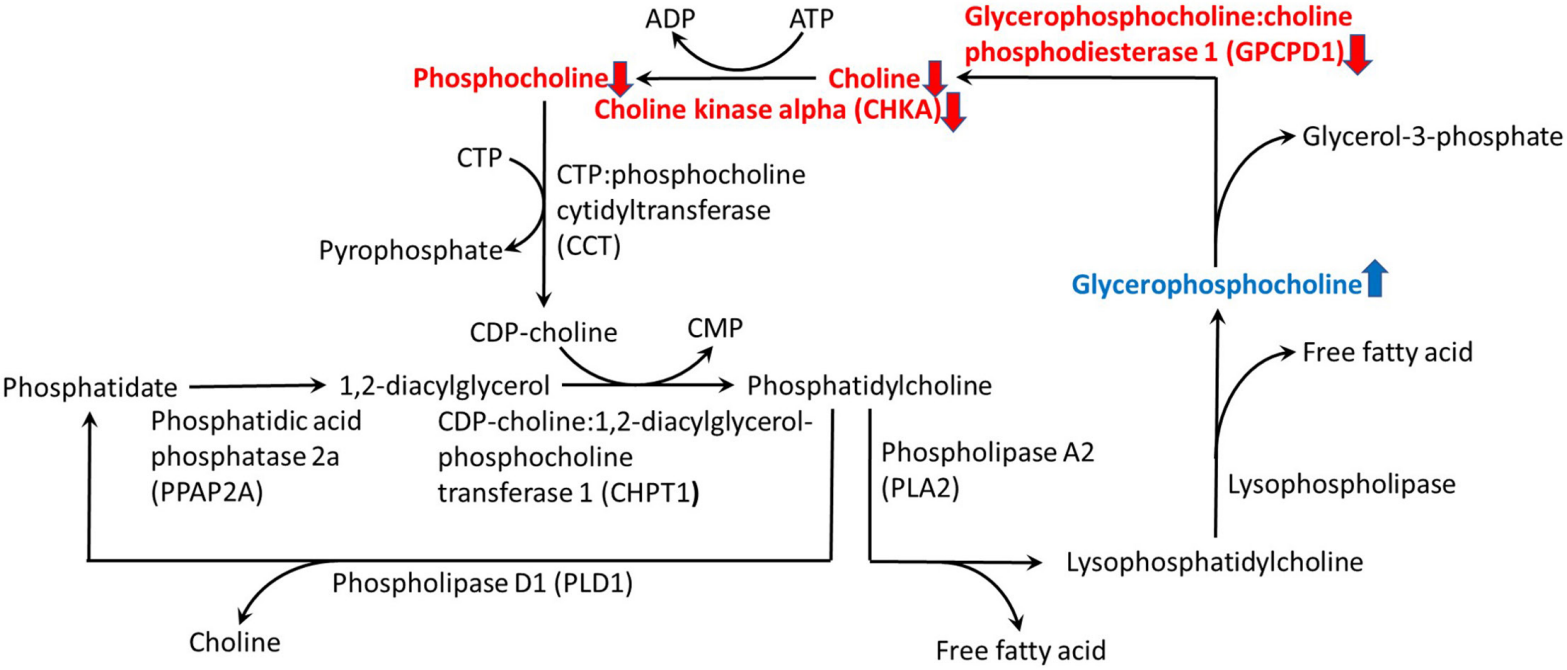
Altered choline metabolism in the brain in Huntington's disease patients. GPCPD1 is significantly downregulated in the striatum and cortex, leading to decreased choline and phosphorylcholine and increased glycerophosphocholine.

In the present study, we detected decreased G*pcpd1* gene expression in the striatum and reduced Gpcpd1 protein expression in both the striatum and cortex in R6/2 HD‐transgenic mice. Gpcpd1 protein expression in the brains of R6/2 HD mice progressively decreased beginning at the age of 6 weeks, indicating that this process occurs at the early stage of HD, when the phenotype began to manifest and may contribute early neuronal dysfunction. Subsequent metabolite analysis indicated reduced levels of choline and phosphorylcholine and increased glycerophosphocholine in the striatum, likely due to the diminished expression of Gpcpd1. Consistent with these findings, lower GPCPD1 protein and *GPCPD1* gene expression levels were confirmed in both the striatum and cortex of postmortem brains of HD patients. These results suggest that reduced GPCPD1 may play a key role in the pathogenesis of HD and that therapeutics restoring choline may have beneficial effects (Figure 7). Citicoline administration to R6/2 mice significantly improved motor performance and reduced apoptosis and lipid peroxidation in the striatum and cortex. Metabolomic profiling revealed partial restoration of metabolic patterns in both the striatum and cortex, highlighting the potential therapeutic impact of citicoline in HD, possibly through normalization of disturbed metabolism.

GPCPD1 plays a pivotal role in the choline‐phosphatidylcholine metabolic pathway, catalyzing the conversion of glycerophosphocholine to phosphocholine and choline (Corda et al., 2014). This enzyme is particularly important for regulating choline homeostasis (Marcucci et al., 2010). A reduction in phosphatidylcholine synthesis by blockage of choline kinase activity has been shown to impede neurite outgrowth in N2a neuroblastoma cells (Domizi et al., 2019; Paoletti et al., 2016). Similarly, we also show reduced *choline kinase A* mRNA expression in striatum of HD mice. Choline also plays a critical role in the synthesis of acetylcholine (Loffelholz et al., 1993), which is impaired in R6/2 mice (Farrar et al., 2011; Holley et al., 2015; Smith et al., 2006; Vetter et al., 2003). Furthermore, choline can donate its methyl group and participate in epigenetic modifications, such as DNA and histone methylation (Bekdash, 2018). Altered DNA methylation patterns have been found at promoter, proximal, and distal regulatory regions associated with neurogenesis and neuronal differentiation genes in the striatal cells of *Hdh* ^*Q111*^ knock‐in HD mice (Ng et al., 2013). Reduced enrichment of trimethylated lysine 4 of histone H4 (H3K4me3) has been described in both the cortex and striatum of R6/2 mice and HD postmortem human brains (Vashishtha et al., 2013). These findings suggest that GPCPD1 deficiency and altered choline metabolism may play a role in HD pathogenesis. Further research into the connections between GPCPD1 and choline metabolism and epigenetic modifications could provide potential insights into the pathogenesis of HD.

In addition to its role in choline‐phosphatidylcholine synthesis, GPCPD1 likely has other physiological functions. Muscle‐specific knockout of *Gpcpd1* leads to glucose intolerance (Cikes et al., 2023). Under hypoxic conditions, GPCPD1 undergoes depalmitoylation and relocates to the outer mitochondrial membrane (Liu et al., 2023). These mitochondrion‐localized GPCPD1 proteins interact with voltage‐dependent anion channel 1, promoting mitophagy by enhancing the anchoring of phosphatase and tensin homolog‐induced kinase 1 (PINK1)/parkin RBR E3 ubiquitin protein ligase (PRKN)‐dependent ubiquitination (Liu et al., 2023). Mitophagy impairment has been observed in HD striatal cells derived from *Hdh* ^*Q111*^ knock‐in mice and a Drosophila model of HD (Khalil et al., 2015). These findings suggested that GPCPD1 deficiency in the brains of R6/2 mice and HD patients may contribute to mitophagy dysfunction. Notably, the overexpression of a truncated form of GPCPD1 in muscles results in muscle atrophy, suggesting that GPCPD1 plays a regulatory role in muscle differentiation (Okazaki et al., 2010). Untargeted metabolomic profiling of skeletal muscle from aged mice revealed a significant increase in glycerophosphocholine levels, which may indicate that Gpcpd1 expression is decreased during aging (Houtkooper et al., 2011). However, further investigations are needed to gain comprehensive understanding of the role of GPCPD1 in HD pathogenesis.

Citicoline, also called cytidine‐5′‐diphosphocholine (CDP‐choline), is a choline source and can increase plasma choline levels in both rodents and humans (Lopez et al., 1987; Sarkar et al., 2012). However, only a minor fraction of the choline dose provided through citicoline enters the brain. Positron emission tomography studies using the [11C]choline radiotracer have shown that less than 0.1% of the tracer is taken up by the cortex and cerebellum in rats and humans (Tolvanen et al., 2010). Importantly, citicoline does not induce cholinergic intoxication, unlike equimolar doses of choline (Agut et al., 1983). Magnetic resonance spectroscopy studies have shown either a decrease or no change in choline levels in the brains of both old and young human subjects receiving citicoline treatment (Babb et al., 1996). This finding suggested alternative mechanisms for its neuroprotective effects. Citicoline is involved in the synthesis of phospholipids in cell membranes and phospholipids serve as a main source of several lipid mediators (Horibata & Sugimoto, 2021). Citicoline is integrated into a larger metabolic network and its interruption can affect the distribution of lipid‐related metabolites in several pathways (Fagone & Jackowski, 2013). Although, in our study, citicoline administration did not increase brain choline or phosphocholine levels, the comprehensive lipidomics analysis emphasizes the metabolic impact of citicoline treatment. Both positive and negative ion models of lipidomics highlight the effects of citicoline treatment on the lipid metabolism in the cortex and striatum of R6/2 mice. The lipid alterations by citicoline may contribute to the phenotypic improvements of the HD mice. However, the individual identification of changed lipid metabolites should be investigated further in the future to know the in‐depth mechanism of citicoline treatment for HD.

Importantly, citicoline treatment significantly reduced the level of MDA, a marker of lipid peroxidation, in both the striatum and cortex. This finding aligns with known mechanisms of citicoline effects, including the suppression of hydroxyl radical generation (Adibhatla & Hatcher, 2003). Several studies have revealed that nuclear factor erythroid 2‐related factor 2 (NRF2) regulates redox homeostasis in HD and other neurodegenerative diseases (Calabrese et al., 2010; Chang & Chen, 2024). Studies on *Hdh* ^*Q111*^ knock‐in mice demonstrate reduced NRF2 activity and increased oxidative stress in striatal cells (Jin et al., 2013). Overexpression of mutant *HTT* in PC12 cells reduces the expression of NRF2 and its responsive proteins such as GCLC, GSTA4, NQO1, GSTA6, and TXNRD1, which leads to increased oxidative damage (van Roon‐Mom et al., 2008). Furthermore, citicoline prevents the activation of phospholipase A2 (Adibhatla & Hatcher, 2003), which is upregulated in the peripheral blood of HD patients (Cheng et al., 2016). Citicoline also increases sirtuin 1 (SIRT1) levels in rat brains (Hurtado et al., 2005). The SIRT1 inhibitor sirtinol abolishes the neuroprotective effect of citicoline in rats with brain ischemia, suggesting that citicoline may exert its neuroprotective effects by enhancing SIRT1 expression (Hurtado et al., 2013). Notably, knockout of neuronal SIRT1 accelerates neurodegeneration, whereas SIRT1 overexpression extends survival, reduces striatal atrophy, and decreases aggregate burden in R6/2 mice (Jeong et al., 2011). Further research is essential to provide comprehensive mechanistic explanations for the neuroprotective effects of citicoline.

This study revealed the potential contribution of GPCPD1 deficiency in the choline metabolism pathway to HD pathogenesis and shed light on the potential therapeutic effects of citicoline in HD by normalizing metabolic patterns (Figure 7). However, the study shows the limitation of its reliance on animal models, primarily R6/2 mice, which may not fully represent the complexity of HD in humans. Although transcriptional dysregulation is one of pathogenic mechanisms underlying HD (Malla et al., 2021), how *GPCPD1* and GPCPD1 expression are reduced remains to be further investigated. The mechanistic explanations for the alterations in the choline‐phosphatidylcholine pathway in HD and the neuroprotective effects of citicoline remain to be further investigated. Future studies, including mechanistic studies to elucidate the precise role of GPCPD1 deficiency and altered choline metabolism in HD and clinical trials to assess the efficacy and safety of citicoline as an HD treatment, are needed to address these limitations.

## 4 METHODS

### 4.1 Animals, transgenic R6/2 HD mice

Heterozygous R6/2 mice were obtained from Jackson Laboratories (Bar Harbor, ME, USA) and mated to female control mice (B6CBAFI/J) (Mangiarini et al., 1996). The offspring were identified by PCR genotyping of tail DNA using the following primers: 5’‐CCG CTC AGG TTC TGC TTT TA‐ 3′ and 5’‐GGC TGA GGA AGC TGA GGA G‐ 3′. All animals were housed at the Chang Gung Memorial Hospital, Animal Care Facility, with unlimited access to water and breeding chow (PicoLab® Rodent Diet 20, PMI® Nutrition International, St. Louis) under a 12‐h light/12‐h dark cycle. Body weights were recorded daily. Animal experiments were performed under protocols approved by the Chang Gung Memorial Hospital, Animal Care and Utilization Committee, Taiwan.

### 4.2 R6/2 HD mice treated with citicoline

Mice were randomly divided into 3 groups. Twenty R6/2 HD mice were treated with citicoline, and another 20 R6/2 HD mice were treated with vehicle (normal saline). A group of 20 wild‐type (WT) littermates were treated with vehicle for comparison. The R6/2 mice treated with citicoline were injected intraperitoneally daily with 500 mg/kg citicoline dissolved in normal saline beginning at 5 weeks of age until sacrifice. All mice were anesthetized with sodium pentobarbital and sacrificed at the age of 12 weeks. Rotarod performance, body weight, blood sugar, untargeted metabolomics, and levels of choline, phosphorylcholine, glycerophosphocholine, B cell lymphoma 2 (Bcl2), and malondialdehyde (MDA) in different brain regions were examined to test the efficacy of citicoline.

### 4.3 Rotarod test

Mouse motor coordination was assessed using a rotarod apparatus (UGO BASILE, Comerio, VA, Italy) at an accelerating speed (3–30 rpm) over a period of 6 min. The animals were pretrained for one trial at an accelerating speed (3–30 rpm) for 5 min, 2 days before the real test, to allow them to become acquainted with the rotarod apparatus. Each mouse was tested for a maximum of 6 min per trial, with 3 trials per day and an interval of 30 min between trials. The means of the 3 trials were used for comparisons between groups. The latency to fall was automatically recorded. The mean performance of the 3 trials for each animal was used for the analysis. The mice were tested for rotarod performance, body weight, and blood sugar twice a week every week beginning at 5 to 12 weeks of age.

### 4.4 Quantitative real‐time polymerase chain reaction (qRT–PCR)

Total RNA was extracted from the striatum of R6/2 mice (heterozygous, *n* = 14–20) and WT littermates (*n* = 14–20) at 7–13 weeks of age or from frozen postmortem human brain tissues (striatum and cortex) using TRIzol (Invitrogen, Carlsbad, CA, USA) and reverse transcribed to cDNA using the SuperScript III First‐Strand cDNA Synthesis System (Invitrogen, Carlsbad, CA). qRT–PCR results for mouse tissues were generated using a TaqMan 5′‐nuclease assay (assay ID: Mm01333968_m1 for *glycerophosphocholine phosphodiesterase 1* (*Gpcpd1*), Mm00442759_m1 for *choline kinase alpha* (*Chka*), Mm04213225_s1 for *choline kinase beta* (*Chkb*), Mm00477016_m1 for *phosphatidic acid phosphatase 2a* (*Ppap2a*), Mm01289339_m1 for *phospholipase D1* (*Pld1*), Mm00522694_m1 for *choline phosphotransferase 1* (*Chpt1*), and Mm00607939_s1 for *beta‐actin* (*Actb*), Thermo Fisher Scientific, Waltham, MA). The qRT–PCR results for human brain tissues were generated using a TaqMan 5′‐nuclease assay (assay ID: Hs00325631_m1 for *GPCPD1*; Hs99999903_m1 for *beta‐actin* (*ACTB*)). The ABI 7900HT Sequence Detection System (Applied Biosystems, Foster City, CA) was used for qRT–PCR. The reactions included cDNA from 100 to 150 ng of RNA, 900 nM of each primer, and 100 nM of each probe, along with Universal PCR Master Mix (Applied Biosystems, Foster City, CA). Each sample was assessed in duplicate. The cycle threshold (CT) in each reaction was set in the linear range. Relative expression values were normalized to those of *Actb* or *ACTB*. Relative gene expression was calculated using the 2ΔCT method, ΔCT = CT (*Actb* or *ACTB*)—CT (target gene). The CTs of *Actb or ACTB* across different samples ranged between 20 and 22.

### 4.5 Western blot analysis

Total proteins from tissue were extracted using lysis buffer containing 10 mM Tris–HCl (pH 7.5), 150 mM NaCl, 5 mM EDTA (pH 8.0), 0.1% SDS, 1% sodium deoxycholate, 1% NP‐40, and a protease inhibitor mixture (Sigma). The proteins (20 μg) were heated in a boiling water bath for 10 min and then fractionated by 10% SDS‐polyacrylamide gel electrophoresis. The fractionated protein samples were transferred onto a nitrocellulose membrane (Cytiva, Washington, DC, USA), and nonspecific binding was blocked in 5% nonfat dry milk overnight at 4°C. After blocking, the blots were probed with primary antibodies against Gpcpd1 (1:2500 dilution; Abcam, Cambridge, UK), Bcl2 (1:1000 dilution; Millipore Sigma, Burlington, MA, USA), and Actb (1:15000 dilution; Millipore Sigma, Burlington, MA, USA) at 4°C overnight. After extensive washing, the immune complexes were detected by horseradish peroxidase‐conjugated goat anti‐rabbit (1:2000; Cell Signaling, MA, USA) or goat anti‐mouse IgG (1:2500 dilution; Thermo Fisher, MA, USA) antibodies and chemiluminescence substrate (Millipore Sigma, Burlington, MA, USA). The protein bands were quantified by ImageJ (National Institutes of Health and the Laboratory for Optical and Computational Instrumentation, NIH‐LOCI, WI, USA).

### 4.6 Postmortem brain sections

Postmortem brain tissues and sections from HD patients and non‐HD controls (Table S1) were obtained from the National Institutes of Health NeuroBioBank supported by the National Institute of Mental Health, the National Institute of Neurological Disorders and Stroke, the Eunice Kennedy Shriver National Institute of Child Health and Human Development, the National Institute on Aging (NIA), and the National Institute on Drug Abuse.

### 4.7 Immunohistochemistry analysis of human brain sections

Formalin‐fixed paraffin‐embedded human brain sections were dewaxed, rehydrated, and washed with phosphate‐buffered saline (PBS), followed by treatment with 3% hydrogen peroxide for 10 min. Brian sections were blocked with 3% normal goat serum (Gibco #16210064; Thermo Fisher, MA, USA) in PBS at room temperature for 2 h, followed by incubation with a primary antibody against GPCPD1 (1:20; HPA039556; Merck, Darmstadt, Germany) in 1% bovine serum albumin (BSA) in PBS in a humidified chamber at 4°C overnight. The tissue sections were washed with PBS, incubated with Alexa Fluor™ 594 donkey anti‐rabbit secondary antibody (1:400; A21207; Thermo Fisher, MA) and Alexa Fluor™ 488 goat anti‐mouse secondary antibody (1:500; A11209; Thermo Fisher, MA, USA), and counterstained with 4′,6‐diamidino‐2‐phenylindole (1:2000; ENZ‐52404; Enzolifesciences, NY, USA) in 1% BSA at room temperature for 2 h. Finally, the sections were washed with PBS. All images were visualized by confocal microscopy (Leica TCS SP8 X, Leica, Wetzlar, Germany). The fluorescent densities were quantified by ImageJ (NIH‐LOCI).

### 4.8 Immunohistochemistry analysis of R/2 mouse brain sections

Mouse brains were fixed with 10% formalin, embedded in optimal cutting temperature compound, and cut into 20 μm sections using a cryostat, followed by washing 3 times with PBS. Mouse brain floating sections were treated with 0.2% Triton 100 for 5 min and blocked with 3% normal goat serum (NGS; Gibco #16210064; Thermo Fisher Scientific, MA, USA) in 0.1 M phosphate buffer (PB) at room temperature for 2 h. Floating mouse brain sections were incubated with a primary antibody against MDA (1:250; ab6463; Abcam, Cambridge, UK) in 1% NGS in 0.1 M PB in a humidified chamber at 4°C overnight. The tissue sections were washed with PBS, incubated with Alexa Fluor™ 488‐conjugated goat anti‐mouse secondary antibody (1:4000; A11209; Thermo Fisher, MA, USA) and Alexa Fluor™ 594‐conjugated donkey anti‐rabbit secondary antibody (1:400; A21207; Thermo Fisher, MA, USA), and counterstained with DAPI (1:8000; ENZ‐52404; Enzolifesciences, NY, USA) in 1% NGS at room temperature for 1 h. Finally, the sections were washed with 0.1 M PB. All images were visualized by confocal microscopy (Leica TCS SP8 X, Leica, Wetzlar, Germany). The fluorescent densities were quantified by ImageJ (NIH‐LOCI).

### 4.9 Ultra‐performance liquid chromatography–mass spectrometry/mass spectrometry (UPLC–MS/MS) quantification of choline, phosphocholine, and glycerophosphocholine in the R6/2 mouse brain

Mouse brain tissue (striatum) was homogenized and dissolved in 0.5 mL of 90% methanol on ice. The suspension was incubated on ice for 5 min and then centrifuged at 12,000 rpm for 10 min at 4°C. The supernatant was transferred to a new vial and completely dried under nitrogen gas. The residue was dissolved in 500 μL of 50% acetonitrile containing an internal standard for UPLC–MS/MS analysis.

All the organic solvents and mobile phases used were prepared using LC/MS‐grade chemicals and were purchased from Sigma Aldrich. Liquid chromatographic separation was achieved on an ACQUITY UPLC BEH Amide column (1.7 μm, 2.1 × 150 mm; Waters, Milford, MA) using an ACQUITY TM Ultra Performance Liquid Chromatography system (Waters). The column was maintained at 45°C, and the flow rate was set at 0.4 mL/min. Solvent A was 0.1% formic acid in water, and solvent B was acetonitrile containing 0.1% formic acid. The samples were eluted from the LC column using a linear gradient: 0–0.1 min, 99% B; 0.1–7 min, 99%–30% B; 7–7.2 min, 30%–99% B; and 7.2–10 min, 99% B for re‐equilibration. Mass spectrometry was performed on a Waters Xevo‐TQMS (Waters) operated in electrospray ionization positive ion mode with multiple‐reaction‐monitoring mode. Single analyte standards were dissolved in a mixture of water/methanol 50:50 (v/v) and then infused at a flow rate of 10 μL/min for tuning purposes, after which the major MS/MS fragmentation patterns of each analyte were determined. The optimized parameters were as follows: the desolvation gas flow rate was set to 800 L/hr at 500°C, and the temperature was set at 150°C. The cone gas flow rate was 150 L/hr. The capillary voltage was 3000 V.

### 4.10 Lipidomic profiling of R6/2 mouse brains using ultra‐performance liquid chromatography‐quadrupole/time‐of‐flight mass spectrometry (UPLC/Q‐TOF‐MS)

Mouse brain tissue (from the striatum and cortex) was weighed and homogenized with 0.5 mL of 90% methanol on ice. The samples were subsequently centrifuged at 12,000 rpm for 10 min at 4°C. The supernatant was transferred to a microtube. The remaining sample was extracted again with 0.5 mL of 90% methanol and was centrifuged as described above. The supernatants were combined and dried under nitrogen gas. The residue was dissolved in solvent (isopropanol:acetonitrile:water = 2:1:1) according to the weight of the tissue and analyzed via UPLC/Q‐TOF‐MS analysis. Each sample was analyzed in triplicate.

All the organic solvents and mobile phases used were prepared using LC/MS‐grade chemicals and were purchased from Sigma Aldrich. Chromatographic separation was performed on a Waters ACQUITY BEH C18 column (2.1 mm × 100 mm × 1.7 μm; Waters, Milford, CT, USA). The column temperature was maintained at 60°C. Mobile phase A was acetonitrile/water (40:60, v/v) with 10 mM ammonium formate, and mobile phase B was isopropanol/acetonitrile (90:10, v/v) with 10 mM ammonium formate. The flow rate was 0.45 mL/min, and the solvent gradient was as follows: 0–10 min, 40%–99% solvent B; 10–10.1 min, 99%–40% solvent B; 10.1–12 min, 40% solvent B; and then re‐equilibrated for 1.9 min. Mass spectrometry was performed on a Waters Q‐TOF‐MS (Xevo G2XS, Waters MS Technologies, Manchester, UK) operating in positive or negative ion mode. The desolvation gas was set at 900 L/h at a temperature of 550°C; the source temperature was set at 120°C. The capillary voltage was set to 2500 and 2000 V in the positive and negative ESI modes, respectively. The sample cone was set at 25 V. The mass range was 100–1700 m/z, and the scan time was 0.1 s. Leucine encephalin was used as the lock mass standard (an [M + H]^+^ ion at 556.2771 Da in positive mode; an [M + H]^−^ ion at 554.2615 Da in negative mode).

MS data were processed using MassLynx V4.1 and Progenesis QI software (Waters Corp., Milford, Massachusetts, USA). Significant metabolites were subjected to a database search using an in‐house database and the Human Metabolome Database (http://www.hmdb.ca/).

### 4.11 Statistical analysis

All the statistical analyses were performed using IBM SPSS Statistics (IBM, Chicago, IL, USA). Gene expression levels and metabolite concentrations were presented as the means ± standard errors of the means for continuous variables and counts (percentages) for categorical variables. Group comparisons were performed using the Mann–Whitney *U* test for pairwise comparisons and Kruskal–Wallis one‐way ANOVA with Dunn's post hoc test when appropriate.

## AUTHOR CONTRIBUTIONS

Conceptualization, CMC; data curation, KHC and CMC; formal analysis, KHC and CMC; funding acquisition, CMC; methodology, MLC and HYT; project administration, CMC; resources, CMC; supervision, CMC; validation, KHC and CMC; visualization, KHC and MLC; writing—original draft, KHC; writing—review and editing, KHC and CMC. All the authors have read and agreed on the published version of the manuscript.

## CONFLICT OF INTEREST STATEMENT

The authors declare no competing interests.

### DATA AVAILABILITY STATEMENT

The datasets generated during the current study are available from the corresponding author at reasonable request.

## Supporting information


Data S1.
