# Supplementary material for: Dysregulation of choline metabolism and therapeutic potential of citicoline in Huntington's disease
Source: Aging Cell. 2024 Aug 14;23(11):e14302. doi: 10.1111/acel.14302 (PMC11561662; doi:10.1111/acel.14302)
Supplement: Supplementary file 1 — Data S1. [file ACEL-23-e14302-s001.docx]

**TABLE S1**  Brain tissue specimens from NIH NeuroBioBank (USA) for immunohistochemical staining and qRT‒PCR analysis

| Case number | Diagnosis | Tissue | Age (years) | Sex | PMI (hours) | Assay |
| --- | --- | --- | --- | --- | --- | --- |
| 4374 | HD | Striatum  Cortex | 48 | Male | 27.15 | IHC  qRT‒PCR |
| 4393 | HD | Striatum  Cortex | 68 | Male | 25.61 | IHC  qRT‒PCR |
| 7167 | HD | Striatum  Cortex | 50 | Female | 17.33 | IHC  qRT‒PCR |
| 8034 | HD | Striatum  Cortex | 69 | Female | 14.12 | IHC  qRT‒PCR |
| 10086 | HD | Striatum  Cortex | 69 | Female | 26.3 | IHC  qRT‒PCR |
| 11136 | HD | Striatum  Cortex | 69 | Female | 19.13 | IHC  qRT‒PCR |
| 11970 | HD | Striatum  Cortex | 44 | Male | 25.67 | IHC  qRT‒PCR |
| 12836 | HD | Striatum  Cortex | 51 | Male | 21.83 | IHC  qRT‒PCR |
| 17759 | HD | Striatum  Cortex | 56 | Female | 22.37 | IHC  qRT‒PCR |
| 17816 | HD | Striatum  Cortex | 66 | Male | 32.83 | IHC  qRT‒PCR |
| 1447 | Normal control | Striatum  Cortex | 63 | Male | 17.92 | IHC |
| 4888 | Normal control | Striatum  Cortex | 58 | Male | 19.05 | IHC |
| 8112 | Normal control | Striatum  Cortex | 74 | Male | 21.08 | IHC  qRT‒PCR |
| 10728 | Normal control | Striatum  Cortex | 62 | Female | 23.5 | IHC |
| 12237 | Normal control | Striatum  Cortex | 68 | Male | 16.05 | IHC |
| 15548 | Normal control | Striatum  Cortex | 69 | Female | 18.65 | IHC |
| 19232 | Normal control | Striatum  Cortex | 58 | female | 19.38 | IHC |

HD: Huntington’s disease; IHC: immunohistochemistry; PMI: postmortem interval; qRT‒PCR: quantitative real-time polymerase chain reaction


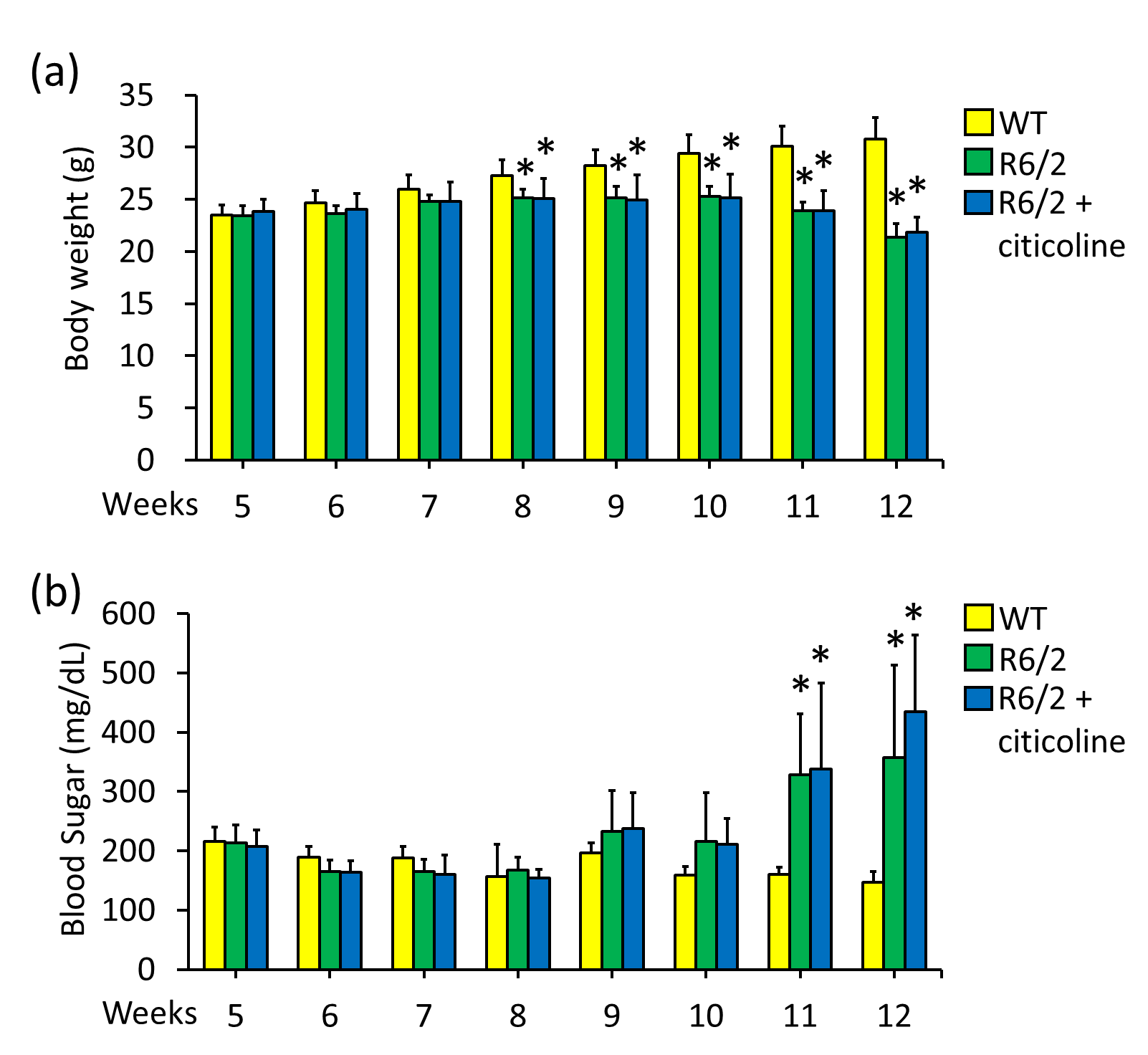


**FIGURE S1** Body weight and blood sugar of R6/2 mice. Body weight (a) and blood sugar (b) of R6/2 mice treated with (n = 20) or without (n = 20) citicoline from 5 to 12 weeks of age compared with wild-type (WT) littermates. **P* < 0.05, R6/2 or R6/2 + citicoline vs WT.
